# Characterization of a Rare Mosaicism in Autosomal Translocation of t(5;21) Using Conventional Cytogenetics and FISH Methods

**DOI:** 10.29252/ibj.24.1.60

**Published:** 2019-07-14

**Authors:** Sadaf Omori Sarabi, Javad Karimzad Hagh, Claudia Behrend, Seyed Behrooz Mohseni, Mitra Ansari Dezfouli, Seyed Khalil Rashidi, Mir Davood Omrani

**Affiliations:** 1 Paresh Pathobiology and Genetics Laboratory, Tehran, Iran;; 2 Praxisgem für Medizinische Genetik, Düsseldorf, Germany;; 3 Department of Neuroscience and Addiction Studies, School of Advanced Technologies in Medicines, Tehran University of Medical Science, Tehran, Iran;; 4 Biotechnology Research Center, Semnan University of Medical Science, Semnan, Iran;; 5 Department of Medical Genetics, Faculty of Medicine, Shahid Beheshti University of Medical Science, Tehran, Iran;; 6 Urogenital Stem Cell Research Center, Shahid Beheshti University of Medical Science, Tehran, Iran

**Keywords:** Partial monosomy 21q, Translocation t(5;21), Unbalanced autosomal chromosome translocation mosaicism

## Abstract

**Background:**

Mosaicism of a normal cell population and an unbalanced autosomal chromosome rearrangement is rarely seen. If the abnormal cell line contributes to a minor part of soma, the phenotype is expected to be normal.

**Case Report:**

We report a 29-year-old woman who had balance chromosomal translocation of 46,XX,t(5;21) with a two-year-old affected girl, characterized by mental retardation, dystrophia, hearing impartment, and dysphagia.

**Methods and Results:**

Cytogenetic investigation revealed a low mosaic unbalanced translocation of 46,XX,t(5;21)/ 46,XX, which was confirmed by FISH analysis. Studying 200 metaphases and interphases of peripheral blood sample revealed 70% partial monosomy of 21q22 and partial trisomy of 5q(35.3) and 30% of normal pattern.

**Conclusion:**

In rare cases such as this study, parents with balanced translocation with no phenotypes may lead to a mosaic unbalanced translocation with abnormal phenotypes in offspring, which underscores the need for prenatal karyotyping and genetics counseling.

## INTRODUCTION

Chromosomal mosaic describes a term that two or more genetically different cell populations present in one individual who has been developed from a single zygote^[^^1^^-^^5^^]^. There are a number of different mechanisms involved in chromosomal mosaicms, including chromosomal abnormalities and DNA mutations. Most somatic mosaicisms are the consequence of post-zygotic errors in recombination or replication. Moreover, whole chromosomal aneuploidies can occur by non-disjunction or anaphase lagging. Imbalances of chromosomal segments can be derived from unrepaired breakages^[^^6^^]^. Whether a mosaism contributes to diseases depends on which tissue is affected and what fraction of the tissue is involved. If the abnormality contributes to small fraction of soma, the phenotype is expected to be normal, but if it includes an extensive part of the soma, it can be associated with variation in phenotypic expression or mental retardation.

Somatic chromosomal mosaicms, including the low level of abnormal cell line and high percentage of normal cells, can be missed by conventional cytogenetics or even recognized as an artifact. In most cases, carriers with the mosaic pattern of chromosomes with no clinical significant will not be investigated.

To date, a few cases of chromosomal mosaicism, including normal and unbalance autosomal translocation cell line, have been published^[^^7^^]^, but no cases of partial trisomy of 5q and partial monosomy of 21q mosaicism have been reported so far. We report a rare inheritance of unbalanced mosaic translocation of a child from a mother with balanced translocation of chromosomes 5 and 21. A strong correlation between the clinical features of the patient and her chromosomal abnormality remains unclear. 

## Case Reports

We present a novel case, a two-year-old affected child characterized by mental retardation, dystrophia, low weight, hearing impairment and dysphasia, who was referred for genetics counseling from a 29-year-old and apparently normal mother.

**Cytogenetics studies**

**Fig. 1:**
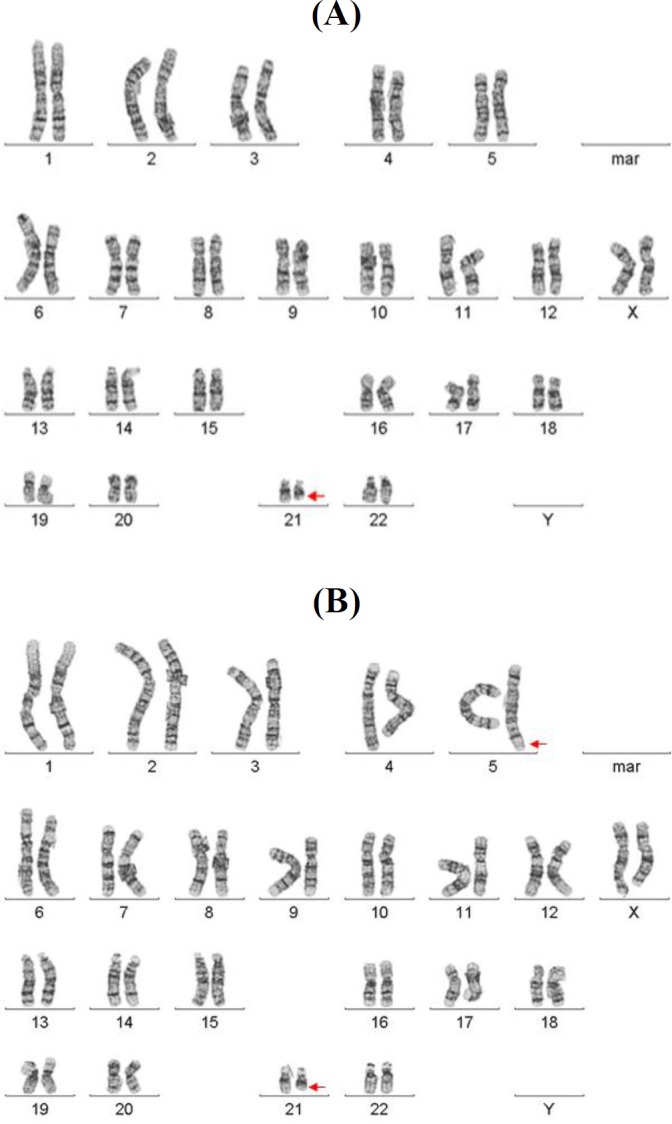
peripheral blood sample karyotype of (A) child with unbalanced translocation of mos46,XX, der(21)t(5;21) (q35.3;q22.1) and (B) mother with balanced translocation of 46,XX,t(5;21)(q35.3;q22.1)

Cytogenetic analysis was performed based on the GTC banding of chromosomes from child’s peripheral blood culture. Analysis revealed in 44 out of 50 metaphases a mosaic unbalanced translocation between the long arm of chromosome 5 and the long arm of chromosome 21. The karyotype of the child was mos46,XX,der(21)t(5;21)(q35.3;q22.1)mat[44]/46,XX [6], resulting in partial monosomy of 21q and partial trisomy of 5q (Fig. 1A). Therefore, parental chromosome analysis was carried out and indicated the chromosome constitution of apparently balanced translocation of 46,XX,t(5;2) on lymphocyte study (Fig. 1B). The patient, therefore, had partial trisomy of 5q and partial monosomy of 21q, which was the result of apparently balanced translocation of chromosome 5q and 12q from her mother.

**Fig. 2:**
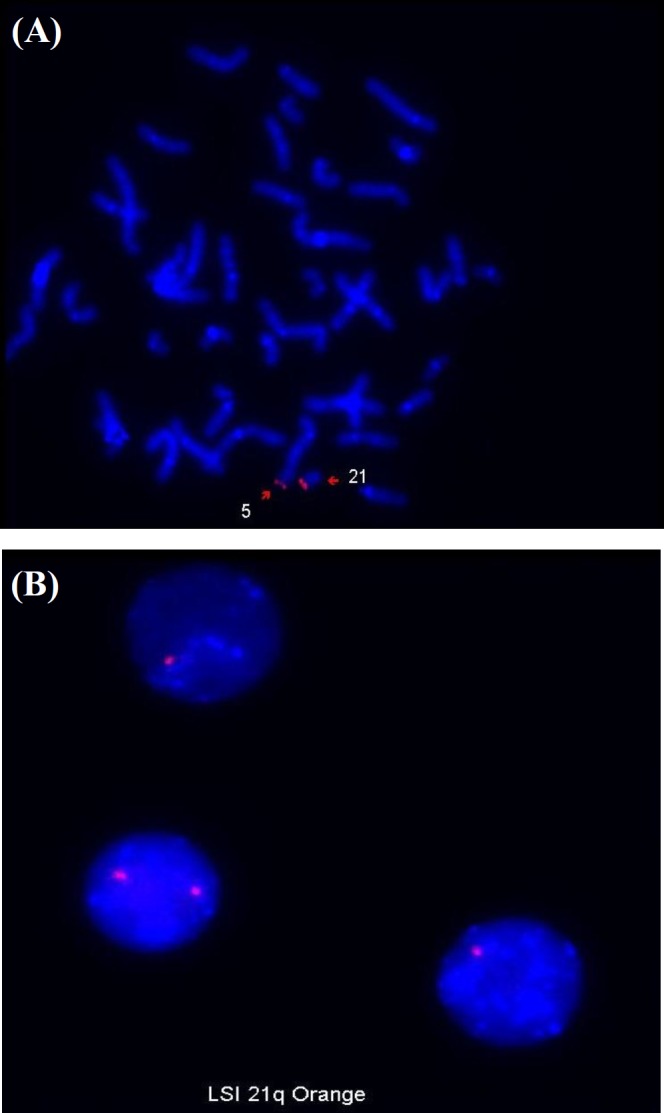
FISH analysis using LSI 21 spectrum orange q22.1.22.1 probe on peripheral blood sample of (A) mother illustrating balance translocation of 46,XX,t(5;21)(q35.3;q22.1) and (B) child illustrating mosaic unbalance translocation of 46,XX,t(5;21)(q35.3;q22.1)

Further FISH analysis was carried out on mother’s peripheral sample using LSI 21 spectrum orange q22.2 FISH-Probe (Vysis), which was distal to the break point. Signals of 50 metaphases confirmed the same balanced translocation pattern as conventional cytogenetic result (Fig. 1A). This method also was conducted for the child mapping in the affected chromosome regions. The study of 200 metaphases and interphases showed 70% partial monosomy 21q and 30% of normal female pattern, which defines the mosaic unbalanced translocation of 46, XX, t(5;21), and also validates the conventional cytogenetic findings (Fig. 2B).

## DISCUSSION

An unbalanced mosaicism of t(5;21) from parent with balanced translocation is extremely rare. A few cases have been reported to date^[^^8^^-^^12^^]^. There are a number of different possible mechanisms that can contribute to initiate this phenomenon: (1) a mitotic exchange of non-homologue chromatids, followed by the loss of one of the translocated chromatids, resulting in an unbalanced and normal cell line; (2) an unbalanced zygote tracked by the loss of the abnormal chromosome, a subsequent monosomy rescue and duplication of the normal chromosome; this would result in an isodisomy for this chromosome^[^^13^^]^; (3) asymmetric 3:1 degree of quadrivalent synapses segregation including the derivative chromosome and two normal combined chromosomes. Loss of a normal chromosome in one cell and loss of the derivative chromosome in other cell will produce two diverse cell populations; (4) chimerism^[^^14^^]^.

The child with mosaic unbalanced translocation long arm of chromosome 5 and long arm of chromosome 21 inherited from her mother with balance translocation is presented here. The patient exhibited a number of clinical symptoms such as mental retardation, dystrophia, low weight, hearing impartment, and dysphasia.

According to literature, trisomy of 5q35 may associate with microcephaly, short stature, developmental delay, and delayed bone maturation. Also, monosomy in long arm of chromosome 21q22 has been reported, with symptoms such as mental retardation, prominent occiput, high nasal bridge, downward slanting eyes, and supernumerary ribs. Since there is a combination of 5q35 trisomy and 21q22 monosomy in the presented case, overlap of both symptoms is recognizable^[^^15^^,^^16^^]^.

In the majority of cases reported in literature, the exact hypothesis or mechanism has not been described for such a “structural chromosomal mosaicism”. However, there is a theory that this translocation can be caused and explained by the asymmetric 3:1 (three chromosomes to one cell and one to the other) degree of quadrivalent synapses in 5;21 reciprocal translocation in maternal meiosis, which leads to two possible eggs pattern, one consists of 22 chromosomes with derivative chromosome 5 (der5) and loss of chromosome 21 (-21), which will probably be lethal after fertilization, and the second is composed of 24 chromosomes including two normal chromosomes 5 and 21 with an extra der21 chromosome, which ends up after fertilization in a embryo with 47,XX,der (21)t(5;21) (q35.3;q22.1),+21. Later on, in post-zygotic events, c two unknown consecutive complex steps (anaphase lagging) may result in two different cell lines, a biparental disomy (46,XX,der(21)t(5;21) (q35.3; q22.1) and a uniparental disomy (46,XX), in a mosaic constellation. According to the literature, the other most likely factor is the embryo in rare instances. 

In rare instances such as this case, parents with balanced translocation with no abnormal phenotype may lead to a *de novo *mosaic unbalanced translocation with abnormal phenotypes in offspring, which underscores the need for an amniocentesis or CVS karyotype, complementary with genetics counseling. These assessments may have valuable outcome.

In conclusion, the characterization of this case illustrates quiet clear that relevant new insight into prenatal phenotypes of rare chromosome conditions can be succeeded only by combing different cytogenetic, molecular cytogenetic findings with the result of ultra-sonographic scanning.

## CONFLICT OF INTEREST.

None declared.
